# Integrating experimental and distribution data to predict future species patterns

**DOI:** 10.1038/s41598-018-38416-3

**Published:** 2019-02-12

**Authors:** Jonne Kotta, Jarno Vanhatalo, Holger Jänes, Helen Orav-Kotta, Luca Rugiu, Veijo Jormalainen, Ivo Bobsien, Markku Viitasalo, Elina Virtanen, Antonia Nyström Sandman, Martin Isaeus, Sonja Leidenberger, Per R. Jonsson, Kerstin Johannesson

**Affiliations:** 1 Estonian Marine Institute, University of Tartu, Mäealuse 14, EE-12618 Tallinn, Estonia; 2 Department of Mathematics and Statistics and Organismal and Evolutionary Biology Research Program, University of Helsinki, FIN-00014 Helsinki, Finland; 3 Centre for Integrative Ecology, Deakin University, 221 Burwood Hwy, Melbourne, Victoria 3125 Australia; 4 Department of Biology, University of Turku, FIN-20014 Turku, Finland; 5 GEOMAR Helmholtz Centre for Ocean Research Kiel, 24105 Kiel, Germany; 6 Finnish Environment Institute, FIN-00251 Helsinki, Finland; 7 AquaBiota Water Research, Löjtnantsgatan 25, SE-11550 Stockholm, Sweden; 8 Ecological Modelling Group, School of Bioscience, University of Skövde, SE-54128 Skövde, Sweden; 9 Department of Marine Sciences – Tjärnö, University of Gothenburg, Tjärnö, SE-45296 Strömstad, Sweden

## Abstract

Predictive species distribution models are mostly based on statistical dependence between environmental and distributional data and therefore may fail to account for physiological limits and biological interactions that are fundamental when modelling species distributions under future climate conditions. Here, we developed a state-of-the-art method integrating biological theory with survey and experimental data in a way that allows us to explicitly model both physical tolerance limits of species and inherent natural variability in regional conditions and thereby improve the reliability of species distribution predictions under future climate conditions. By using a macroalga-herbivore association (*Fucus vesiculosus* - *Idotea balthica*) as a case study, we illustrated how salinity reduction and temperature increase under future climate conditions may significantly reduce the occurrence and biomass of these important coastal species. Moreover, we showed that the reduction of herbivore occurrence is linked to reduction of their host macroalgae. Spatial predictive modelling and experimental biology have been traditionally seen as separate fields but stronger interlinkages between these disciplines can improve species distribution projections under climate change. Experiments enable qualitative prior knowledge to be defined and identify cause-effect relationships, and thereby better foresee alterations in ecosystem structure and functioning under future climate conditions that are not necessarily seen in projections based on non-causal statistical relationships alone.

## Introduction

Global climate change has a remarkable, but still largely unexplored potential to alter both terrestrial and aquatic ecosystems by directly modifying the abiotic environment (e.g. changes in temperature, rainfall, acidity, salinity) and indirectly altering biotic interaction networks^1–3^. This in turn drives the need for ecologists to examine whether and how ecosystems and species within these systems can withstand such rapid environmental changes.

Species distribution responses to climate change are commonly studied using predictive species distribution models (SDMs). These models are often based on finding statistical dependence between environmental and distributional data^4–9^. Even though these models do not rely only on correlations, they are often called correlative since they do not include mechanistic, causal, knowledge on a species’ dependence on its environment or other species^10^. Moreover, an important assumption behind most of them is that relationships between the observed patterns of environment and species distribution will remain unchanged over the study region and time^6^. Due to non-stationarity of ecosystem processes^11–16^ such an assumption seems unrealistic and will likely be violated under future climate conditions, when statistical patterns between current species distributions and the environment are expected to become uncoupled^17,18^. Moreover, under future climate scenarios statistical SDMs are often applied outside the environmental gradient where they have been initially trained^6,10,19,20^ in which case the results may become unreliable^21^. One potentially important aspect that may affect range shifts driven by climate change is the presence of locally adapted populations^22^ with varying potential to respond to shifts in environmental conditions^23^. Local differentiation in tolerance is currently poorly understood but an inclusion of such genetic within-species variation likely improves the model performance.

A fully mechanistic SDM requires extensive data about how species’ unique biology governs their responses to environmental factors^19,24^. In order to overcome the issue of data deficiency, hybrid statistical-mechanistic SDMs offer a pragmatic approach to add key mechanisms to simple statistical predictive SDMs^10,13,25^. Recent studies have highlighted the need to include physiological limits in the form of tolerance to varying environmental conditions^21^. Although these physiological mechanisms play key roles in mediating biotic responses to climate change, current predictive SDMs mostly neglect tolerances of species to environmental factors^26–28^. Various physiological thresholds are known for a number of taxa and can be experimentally tested under elevated stress conditions. Therefore, information from such tolerance experiments, especially when including relevant aspects and levels of possible futures, should be combined with traditional SDM approaches in order to achieve more robust projections of biotic patterns under known climate change scenarios^19,29^. Although it is widely acknowledged that communities are more than the sum of the parts, most SDMs neglect biological mechanisms despite the fact that species interactions often explain unexpected responses to climate change^13,20,30,31^, and most extinctions attributed to climate change to date have involved altered species interactions^32^. Thus, informing statistical SDMs with the species-specific tolerance limits of locally adapted populations, and including the main species interactions into these models, will significantly increase model realism and improve climate change projections^16,20,33,34^.

**Figure 1:**
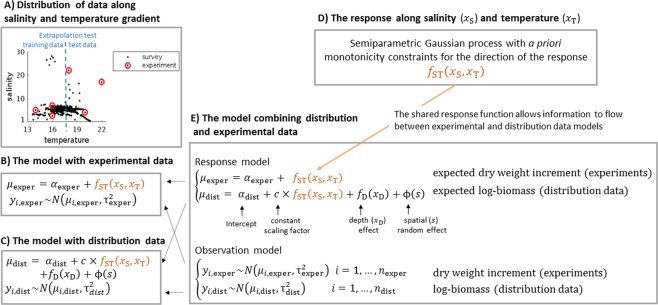
Summary of the noncausal distribution and causal experimental data (**A**) and the SDMs with alternative data (**B**–**E**). Panel E shows the model where both *Fucus* biomass data (distribution) and growth data (experimental) are combined. The key component of the model is the response function along salinity and temperature (**D**) which is shared between the model’s experimental and distribution data components. This allows integrating the information from these two information sources. The rest of the model components are specific to either experimental or distribution data and explain the mean level of data (*intercept*) and the structured (*effect of depth* and *spatial random effect*) and unstructured (*Gaussian noise*) variation in observations not explained by temperature and salinity. When analyzing experimental (**B**) and distribution data (**C**) separately we used only the respective model components from the combined model (**E**) in which case information from only either one of the data sources is used to learn the response along salinity and temperature. The alternative models were compared in interpolation and extrapolation scenario by training the models with experimental data and a subset of the distribution data (training data) and predicting to the left out distribution data (test data). In interpolation tests, distribution data were divided at random and in extrapolation tests the data were divided at 17.5 degrees temperature (blue dashed line in **A**). The *Fucus* presence/absence model follows independently the same hierarchical structure as presented here. The *Idotea* model is otherwise similar but instead of depth it includes the effect of *Fucus* biomass in the distribution data model.

In order to improve the realism of statistical SDMs under climate change projections, we developed a novel semiparametric methodology that can combine qualitative prior information and experimentally defined tolerance levels on species’ response to selected environmental gradients with information obtained from surveyed non-causal distribution data. The methodology extends the state-of-the-art Gaussian process (GP) SDMs^35–37^ under an hierarchical Bayesian approach (Fig. 1). We quantified the current and future distribution patterns of the foundation macroalgal species *Fucus vesiculosus* (*Fucus* hereafter) and its herbivore *Idotea balthica* (*Idotea* hereafter) throughout the Baltic Sea. Importantly, both distributional and experimental data were resolved at sub-regional level (i.e. entrance, central and marginal areas of the Baltic Sea) to account for potential local differentiation in tolerance as well as spatial variation in current and future environmental conditions. Moreover, experiments evaluated the physiological tolerance of *Fucus* and *Idotea* to the projected changes in salinity and temperature as they are the most important regional drivers of future biotic communities in the Baltic Sea^38^.

The Baltic Sea can serve as an excellent case study area to research the consequences of climate change, as climate change signals are visible already now and predicted to be particularly strong in the future^38^. Moreover, due to a faster trajectory of anthropogenic perturbations and the presence of a diverse range of interacting pressures that most coastal areas will experience only in the future, evidence from the Baltic Sea may deliver important insight for the future coastal ocean^39^. Last but not least, the Baltic Sea region is also one of the most intensely studied coastal areas with high data density and many long-term data series. As such our study is a rare demonstration case in which the consequences of contemporary climate change can be realistically assessed in a regional sea. This assessment is supported by a novel developed state-of-the-art modelling method that can be applied beyond coastal habitats and aquatic ecosystems and used not only for climate change but also other purposes.

## Results

To test the importance of experimental versus distribution data in SDMs, we compared our hybrid model that combines experimental and distribution data to corresponding models using only either experimental data or distribution data (Fig. 1). With *Fucus* we modelled separately the occurrence (survival in experiments) and biomass (growth in experiments) using salinity, temperature and depth as predictive environmental covariates. Due to the absence of abundance data at the pan-Baltic scale, we modelled only the occurrence of *Idotea* using salinity, temperature and *Fucus* biomass as predictive environmental covariates. The models included also spatial random effect that explained spatially structured variation due to, e.g., missing covariates. We evaluated and compared the models’ performance in interpolation (projection within the current covariate range) and extrapolation (projection beyond the current covariate range) by dividing the distribution data into training and test set, training the models with the former and measuring their predictive performance on the latter. In interpolation test, data were divided randomly and in extrapolation test they were divided structurally (Fig. 1, Materials and Methods).

### *Fucus* distribution

**Figure 2:**
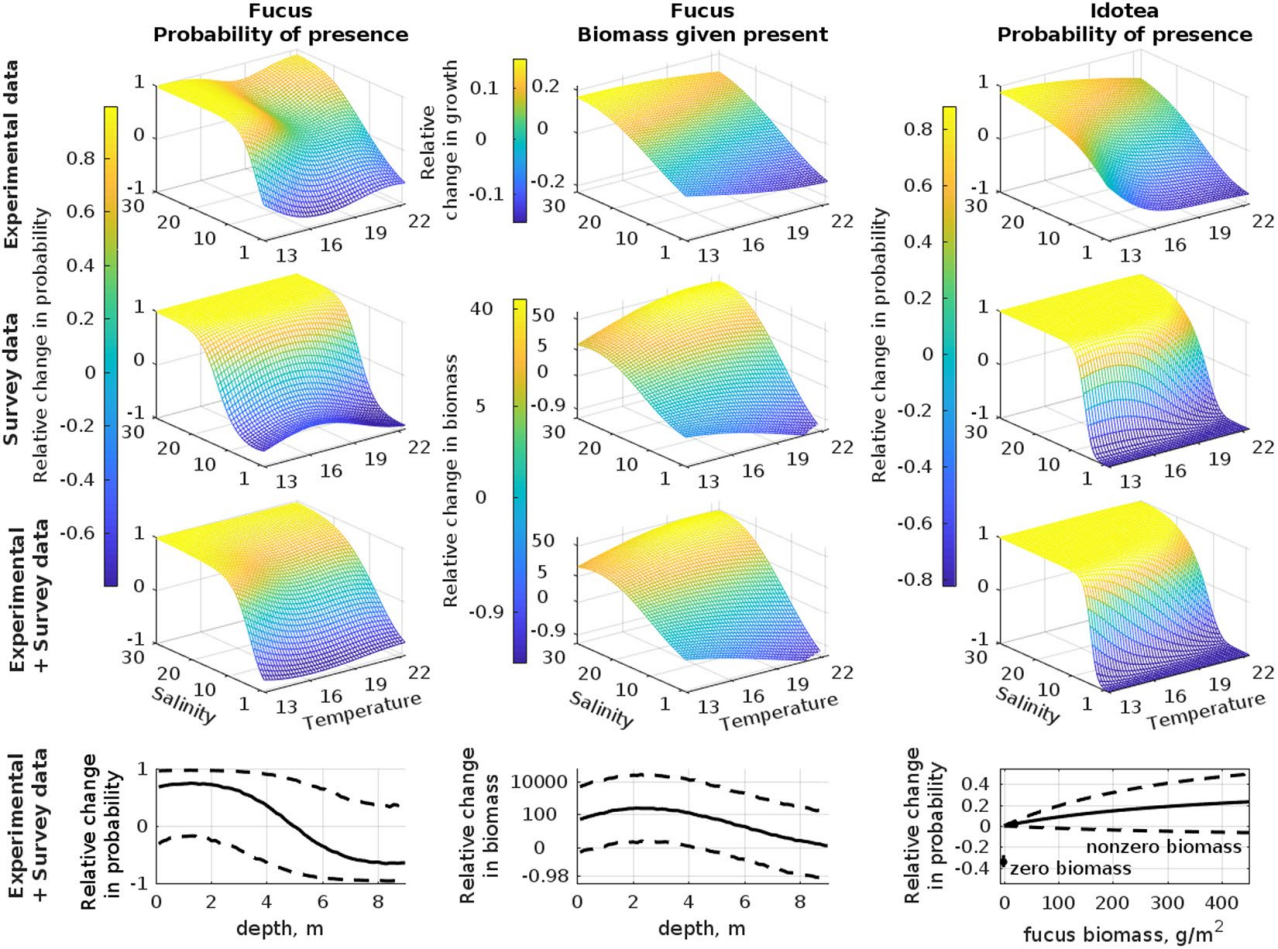
The response of distribution along covariates. The left column shows the relative chance of probability of *Fucus* presence compared to 0.5 probability (an equal chance of occurring or not occurring). The middle column shows the change in *Fucus* growth (experiment) or biomass (survey) given it is present relative to the average growth rate in experimental data or the average biomass in survey data. The third column shows the relative change in probability of occurrence of *Idotea* (compared to 0.5 probability). The surface plots (first three rows) show the posterior median for the three models considered (the experimental data model, the distribution data model and the model combining the experimental and distribution data). The last row shows the posterior median and 95% credible interval along depth (*Fucus* model) and *Fucus* biomass (*Idotea* model).

The explanatory and predictive power of SDMs depended on the type of input data. The *Fucus* SDMs that only included distribution data or combined distribution and experimental data performed equally well in explaining training data (Table 1) and predicting test data in interpolation and extrapolation (Supplementary Table 1). The SDM that included only experimental data, however, yielded poorer interpolation projections but practically equally good extrapolation projections as the other SDMs. Salinity and temperature explained about 30 to 60% of the total explained variability in the (expected) occurrence of *Fucus* but only 20% of the total explained variability in its (expected) biomass (Table 1). Salinity had stronger effects than temperature in these models with temperature having only marginal effects on the probability of occurrence and a small contribution to the growth rate and biomass of *Fucus* (Fig. 2). Water depth had important effects in both models. The spatial random effects explained approximately 20–40% of the total variation and had long spatial correlation length indicating that there is strong regional variation in the distribution of *Fucus* not explained by salinity, temperature and depth.

**Table 1:** Models’ explanatory power and partitioning of variation in biomass, growth and probability of occurrence to different components.

Species	Data source (model)	Explanatory power		Variation partitioning (occurrence/biomass)			
		Tjur-R^2^ (occurrence)	R^2^ (biomass)	Joint effect of temperature and salinity	Effect of depth	Effect of *Fucus* biomass	Spatial random effect
*Fucus*	Experiments	0.47	0.50				
	Distribution	0.42	0.52	0.62/0.20	0.18/0.35		0.20/0.45
	Distribution + Experiments	0.39	0.52	0.30/0.19	0.34/0.39		0.37/0.42
*Idotea*	Experiments	0.09					
	Distribution	0.59		0.63/		0.08/	0.29/
	Distribution + Experiments	0.66		0.58/		0.09/	0.33/

Explanatory power is measured by Tjur-R^2^ (occurrence models) and R^2^ (biomass and growth models) statistics, which measure how well the models explain the training data (n = 2000). The variation partitioning summarizes what proportion of the total variation in expected biomass, growth and expected occurrence (in log odds ratio scale) over the data points is explained by different model components.

Both experimental and distribution data yielded similar responses along salinity gradients (Fig. 2). In all SDMs, the probability of occurrence, biomass per substrate area (biomass hereafter) and biomass increment (growth hereafter) of *Fucus* started to increase at around salinity 3–5 psu (there was an earlier start in increase in the experimental and combined data SDMs compared to the distribution data SDM). The response of probability of occurrence along the salinity gradient was  the steepest at low temperatures and got  gradually less steep as temperature increases. The response along temperature was  the strongest at salinities from 20 to 30 psu. In the combined experimental and distribution data SDM the expected effects of temperature and salinity were  a compromise between the effects in the experimental and distribution data SDMs. In the combined data SDM the response along salinity was  steeper than in the distribution data model and the response along temperature was  similar in shape to the distribution data model.

### *Idotea* distribution

All *Idotea* SDMs were similar in terms of their predictive performance and they outperformed the *Fucus* SDMs (Supplementary Table 1). The joint effect of salinity and temperature explained more than half of the total variation in occurrence. However, most of the variation in this joint effect was attributable to salinity with its strongest effects in SDMs including distribution data. The response along temperature was negligible. The response along salinity was clearly positive in all *Idotea* SDMs (Fig. 2). In addition to direct abiotic forcing, distribution data showed that *Idotea* was also sensitive to changes in the biomass of its host alga *Fucus*. The probability of occurrence of *Idotea* was significantly lower in the absence than in the presence of *Fucus*. Moreover, with high credibility the probability of occurrence of *Idotea* further increased with growing biomass of *Fucus*, and at maximum biomass of host alga the effect of *Fucus* on *Idotea* was about one-third of the effect of salinity. The spatial random effect explained approximately 30% of the occurrence of *Idotea* but there was no clear spatial pattern in the spatial random effect which indicates strong local variability in its occurrence beyond that explained by salinity, temperature and *Fucus* biomass.

### Climate change projections

**Figure 3:**
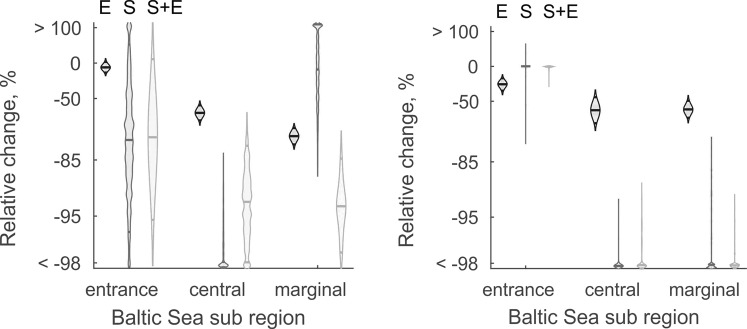
The climate change induced shifts in the *Fucus* mean growth and biomass (left) and the probability of occurrence of *Idotea* (right) in three regions of the Baltic Sea. E denotes experiment, S survey and S + E combined models, respectively. The panels show the relative difference under climate change compared to current conditions (that is: (future-current)/current). In each mark, lines show the posterior expected difference and the 95% credible interval and the shape of the mark indicates the shape of the posterior distribution.

**Figure 4:**
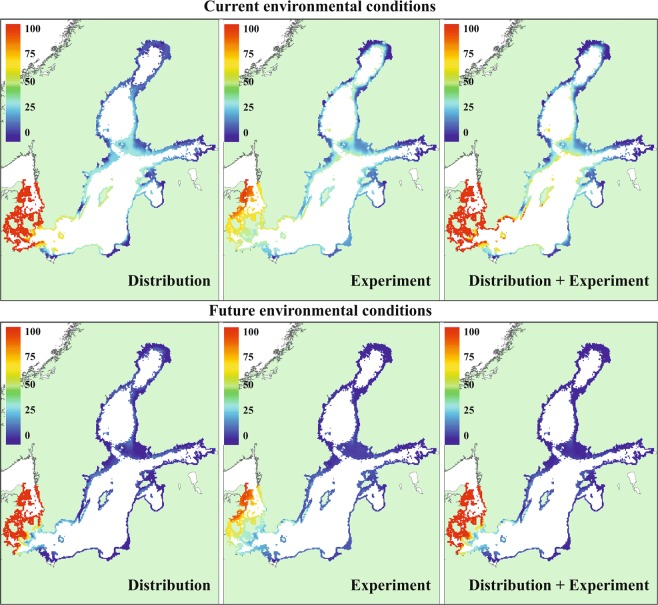
Spatial projection of *Fucus* under current and future environmental conditions. The model including distribution data predicts expected biomass (predicted biomass × probability of occurrence, g m^−2^), the model including experimental data predicts relative growth (% growth of initial value) and the model combining distribution and experimental data predicts expected biomass (predicted biomass × probability of occurrence, g m^−2^).

**Figure 5:**
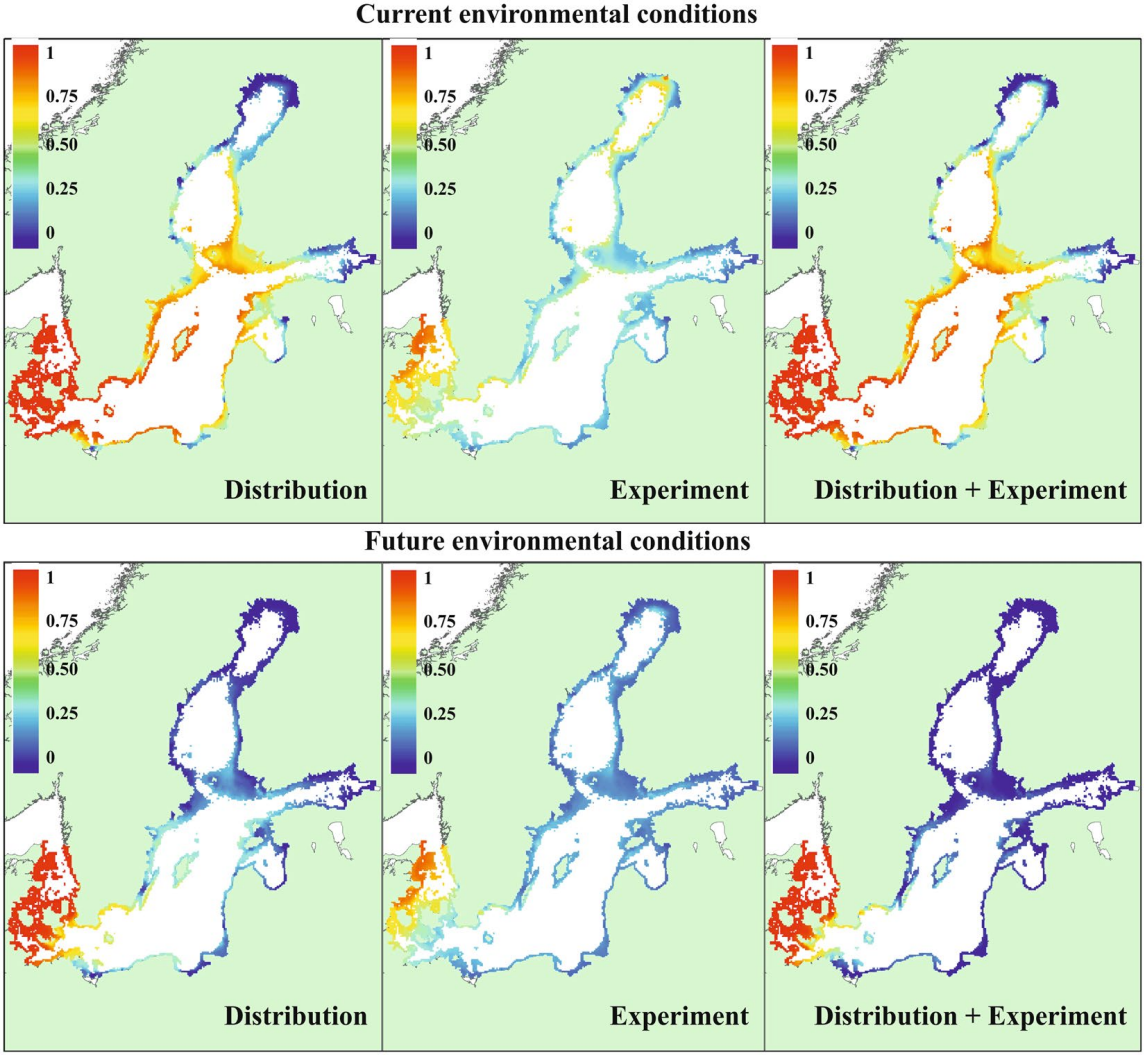
Spatial projection of *Idotea* under current and future environmental conditions. All models predict the expected probability of occurrence.

The probability of occurrence and biomass of *Fucus* as well as the probability of occurrence of *Idotea* had a tipping point at salinities 3–10 psu. The tipping was more radical and happened at lower salinities in cold temperature than warm temperature regions (Fig. 2). Hence, the projections indicate that both *Fucus* and *Idotea* will have a lower probability of survival and probability of occurrence under future climate conditions compared to current environmental conditions (Fig. 3). In addition, *Fucus* will have a lower growth rate (experiment) and biomass (distribution data) under these conditions. The predicted effects of climate change with experimental and distribution data SDMs had similar direction of change but were quantitatively different. In the case of *Fucus*, the predicted relative changes in biomass were larger in the distribution data SDM and in the SDM combining distribution and experimental data compared to relative changes in growth in the experimental data SDM. The former two models had also larger uncertainty related to the projections. The climate induced relative effects on *Idotea* were stronger in the distribution data SDM and in the SDM combining distribution and experimental data compared to the SDM including experimental data only. See Supplementary Figs 4 and 5 for changes in absolute scale. Regardless of models, large areas of *Fucus* habitats would be lost in the central and marginal regions of the Baltic Sea. The projections indicate that the entrance populations of *Fucus* and *Idotea* handle well the future conditions as we did not find differences among current and future climate conditions in any of the responses (Fig. 3). In addition, the models showed that a considerable reduction in the biomass of *Fucus* predicted under future climate conditions would result in a much severer decline of the probability of occurrence of *Idotea* in marginal and central areas than if reduction in *Fucus* biomass was not taken into account (i.e. projections including experimental data only) (Figs 4 and 5).

## Discussion

Here, we built an hierarchical Bayesian approach to combine *a priori* ecological information on expected responses along salinity and temperature gradients with experimental and distribution data. These combined (distribution and experimental data) models performed equally well as the distribution data models when predicting the current distribution ranges of the species and when extrapolating outside the salinity and temperature range in the training data. The models using experimental data only had poorer performance than the models using survey data when predicting the current distribution range but similar performance as the models using survey data when predicting outside current distribution range. The explanatory power, as measured by R^2^ (biomass) and Tjur- R^2^ (occurrence) values (Table 1) was practically similar for all *Fucus* models, whereas in the case of *Idotea* the experimental data model had clearly the worst, and the model combining experimental and distribution data, the best explanatory power.

These results indicate that the experimental and field data contain largely the same information about salinity and temperature tolerance levels at the current environmental conditions. Our field data were very large and therefore the added information from the experiments was small. However, when the size of field data is smaller or when the responses in field and experimental data are different, the experimental data will have larger effect on performance of the combined model.

The experimental data model had worse interpolation performance than the two other models since the current probabilities of occurrence of *Fucus* and *Idotea* are relatively low in marginal and central areas and therefore the prevalence of these species across the study region is smaller on average than predicted by experiments where these species were always originally present. In addition, the distribution of these species is not regulated only by salinity and temperature, which were the environmental variables used in the experiments. In the models with distribution data the spatial random effect allowed adjustment for distribution patterns that could not be explained by temperature and salinity alone and, hence, improved the projections by implicitly modeling the effect of environmental factors not included in the model. These differences between experimental and distribution data partly explain large differences in the Tjur-R^2^ value between *Idotea* experimental data model and the two other *Idotea* models. In the experiments, the survival of *Idotea* was relatively large within all salinity-temperature combinations leading to predictive probabilities near 50%, and hence small Tjur-R^2^, whereas in the distribution data the range in the probability of occurrence of *Idotea* was larger leading to larger Tjur-R^2^.

The distribution data was more evenly spread along the Finnish and Estonian coast as compared to the south-eastern Baltic sea or Swedish coast. Hence, in these sparsely sampled areas we are extrapolating spatially for locations that are far from any sampling station. However, we are not extrapolating as significantly with respect to salinity and temperature, the main factors explaining species distribution in our model, since the distribution data covers well their current gradient (Fig. 6 in the supplement). Due to spatial random effect, our projections are more accurate in locations that are near sampling sites. However, the difference between projected current and future distribution ranges behaves equally well in all regions since in our study these differences are driven by salinity and temperature changes.

Our study clearly demonstrated that climate induced changes are expected to considerably reduce the distribution range of *Fucus* and *Idotea* in the Baltic Sea and their range shrinkage is expected to be similar due to covariation of their distribution. Shifts in salinity displayed a systematically stronger effect than water temperature for both *Fucus* and *Idotea*. Low-salinity regions below 3–5 psu were characterized by reduced occurrence probability, survival, biomass and/or growth. In this study we did not analyse the effect of salinity on reproduction. However, for the studied species, sexual reproduction is possible at salinities above 3.5 psu^40,41^, so our models do not overestimate the future range of these species.

Contemporary climate change, principally caused by human induced global warming poses a threat to marine life^42–44^. Many previous studies have focused on tropical ecosystems with negligible annual temperature fluctuations, and where even a minor temperature increase can lead to regime shifts^45,46^. In contrast, the Baltic Sea shows strong annual fluctuations from subzero during winter to +25 °C in summer, and thereby projected changes in average future temperatures have less severe effects compared to the predicted shift in salinity. Here, both *Fucus* and *Idotea* live within their optimal temperature range and can tolerate much higher average seawater temperatures than predicted by future climate scenarios^47–50^. Nevertheless, the expected increase in temperature may still lead to re-structuring of the Baltic Sea ecosystem through increasing the frequency of heat waves and through multiple synergistic effects of climate change conditions on producer, herbivore and predator trophic levels^47,48,51,52^.

Our *Idotea* models suggest that species interactions are expected to modulate responses induced by contemporary climate change. Most of the species in the Baltic Sea live close to their physiological salinity tolerance limits but different species have different threshold levels. Although *Idotea* tolerate slightly lower salinities than *Fucus*^53^, the range retreats were similar due to the strong preference of *Idotea* for *Fucus* as a host species and consequent covariation of their distribution^54,55^. In fact, our models predicted that the future Baltic Sea has very sporadic occurrences of *Idotea*, mostly associated with the future *Fucus* habitats and as such demonstrated how the availability of habitat and/or resource can narrow the realized niche of a species^56^.

It is important to stress that our modelling study is limited to the provision of *Fucus* habitat for *Idotea* whereas other biotic interactions remained unaccounted for. To some extent the populations of *Idotea* are controlled by fish species; however, the magnitude of such control is not known. Moreover, there is no common agreement on the projections of predator populations to the future Baltic Sea^57,58^. Earlier studies have indicated though, that bottom up processes (i.e. the availability of nutrients and macroalgae) primarily describe the spatial patterns and dynamics of herbivore populations in the study area, even at the marginal low salinity regions^55,59^. Significant effects of *Idotea* on *Fucus* are expected only when populations of the host alga are severely stressed from other disturbances such as strong epiphytic load induced by coastal eutrophication^59,60^. Hence, our results highlight the importance of incorporating biotic interactions in SDM approaches to predict species distribution patterns^10,16,61^. SDMs often neglect species interactions^62^, which impedes the effectiveness of climate change projections, as biotic interactions (e.g. mutualism, competition and predation) may constrain the responses of species^10,61,63,64^.

One potentially important aspect that may affect range shifts driven by climate change is the presence of intra-specific variation in niche space, e.g. ecotypes and locally adapted populations^22^ with varying potential to respond to shifts in environmental conditions^23^. Previous reports suggest that *Fucus* shows inter-regional genetic differentiation^65,66^, although it is poorly known if this variation is correlated with physiological differences. Also for *Idotea* geographic genetic and phenotypic differentations are known^41,67^. Recently, some studies have explored how the presence of locally adapted populations within a species may affect projections from SDMs^68–72^. Although occurrence data may accurately model the present distribution even without any knowledge about locally adapted subpopulations^69^, this may not be the case when SDMs are used to predict future range shifts, e.g. driven by climate change^70,71^. SDM projections may both underestimate^71^ and overestimate^69^ the loss of future habitat when intraspecific variation is neglected. This happens if all individuals are considered to form a single population when training models with data instead of using separate regression fits for each differentiated subpopulation^69^.

In our experiments we sampled *Fucus* and *Idotea* populations along the Baltic Sea, from the entrance to the marginal region^49,50^. Then the species were reared under the current ambient and future conditions projected to occur in the region of origin of the populations. By experimentally quantifying the potential differentiation in tolerance of populations along the salinity and temperature gradients we were able to incorporate realistic responses of locally adapted populations in our joint models.

The models we present here are based on fixed niches so that populations follow the environmental change by range shifts. Another possibility is that populations adapt to tolerate the novel conditions, i.e. the traits providing tolerance evolve through selection by the changing environment^17,73–75^. Thus, a projection of future distribution that takes into account evolutionary responses requires knowledge on heritable genetic variation in tolerance that provides adaptation potential to climate change^73,74^. Unfortunately, scientists seldom know if, or how quickly, the climate-sensitive traits of populations can evolve^75,76^. Our experiment on *Fucus* provided evidence for inter-population variation as well as genotypic variation in the performance responses to future conditions^49^. Thus, in this sense niche-based models may overestimate the predicted range shifts.

Even though an inclusion of the experimental data did not improve the predictive performance of SDMs in our tests, it improves the credibility of our results concerning climate change projections. Since our inference is conditioned on experimental data, we can make more conclusive claims on the cause-effect relationship between temperature, salinity and species distribution than would be possible when including only noncausal species distribution data. The credibility of such interdependencies is of central importance in SDM since it governs how well the model can project species distribution patterns in a set of environmental conditions that we have not yet witnessed but are projected to occur under future climate^7,8,10,35,77^ (see Supplementary Fig. 6).

Conclusive causal experiments on biotic-abiotic interdependencies are possible only in highly simplified settings. When using our methodology, targeted experiments can be planned to inform about the most important abiotic effects and/or interspecific interactions expected to drive the distribution changes. Moreover, we can also infer environmental responses at a wider spectrum of factors than what is possible by using only either of the data sets. One of the strengths of our method is that it is a rather straightforward extension of current SDMs and, hence, easily applicable for other species and statistical SDMs as well. We build our model using semiparametric GP models which have gained increasing interest in ecology in recent years^36,37,78^ because such models allow an easy inclusion of flexible response curves and interaction terms^9,35,37^. However, the hierarchical structure to combine experimental and distribution data could, in principle, be implemented in any statistical SDM, such as generalized linear or additive models^79^. In addition to combining experimental and non-causal distribution data, we extend the existing GP methods by incorporating qualitative prior knowledge on the direction of the response. This is a novel methodological advance in SDMs and is expected to improve the predictive performance of semiparametric SDMs more generally.

However, our method is still purely statistical in the sense that it does not involve any mechanistic description of the cause-effect relationship and it makes only very simplistic assumption concerning interactions between the study species. These components would need further development to better understand the mechnisms behind species distribution changes^6,8,18,20^. One approach towards such methods is, for example, to use functional-traits to explain species responses to their environment^13,29^. Alternatively, our method could also be extended to joint species distribution modeling which would account for interspecific interactions^13,34,80^. Since species response to the environment is typically similar between functionally similar species^13,29^ experiments on only a subset of species may inform about the response of functionally similar species as well.

## Conclusions

By combining qualitative prior knowledge, data from tolerance experiments and field surveys into a single modelling framework, and taking into account the covariation of distributions of closely interacting species, this study greatly contributed towards understanding the effects of climate change on species distribution and enhanced the predictive capacity and robustness of current modelling techniques. Experimentally-derived knowledge allows us to define accurate cause-effect relationships and thereby better foresee alterations in ecosystem structure and functioning under future climate conditions that are not necessarily seen in projections based on statistical (non-causal) SDMs alone. Moreover, an inclusion of region-specific physiological tolerance limits (i.e. local adaptation in tolerance) and plant-herbivore interactions (i.e. species interactions) further increases the predictive power and robustness of model outputs.

As demonstrated in this study, climate change-driven shifts in biota can be dramatic and complex, causing fundamental transitions in ecosystems thereby endangering important ecosystem services. In order to understand trajectories of rapidly progressing global pressures and their consequences, efficient modelling solutions are needed. The methodology developed here is a promising approach to synthetize the often scattered and diverse information on organismal responses to climate change.

## Material and Methods

### Biotic Survey Data

Primary producers are of special importance in most ecosystems, since they fuel other trophic levels with energy and organic matter^81^. Herbivores, in turn, control how much of this energy is transferred to higher trophic levels and thereby define the ecological efficiency of ecosystems^82^. The distribution data of the brown alga *Fucus* and its herbivore *Idotea* were combined from different sources: benthos database of the Estonian Marine Institute, University of Tartu (http://loch.ness.sea.ee/gisservices2/liikideinfoportaal/); the VELMU database, Finnish Environment Institute (http://www.ymparisto.fi/en-US/VELMU); database of the Swedish national monitoring programme (http://sharkdata.se/), benthic inventory data collected by AquaBiota (http://www.aquabiota.se/en/researchservices/inventories-using-underwater-video/)^83^, EurOBIS (http://www.eurobis.org/), EMODnet (http://www.emodnet-biology.eu/portal/), and the HELCOM Red List dataset^84^ (Supplementary Fig. 1).

Altogether 6407 stations from coastal hard bottom habitats of the Baltic Sea, that had quantitative information for both benthic macroalgae and associated invertebrates, were selected for this study (Supplementary Fig. 1). All this dataset had a uniform sampling and sample processing protocol developed for the HELCOM COMBINE programme^85^. The included stations were sampled from June to August between 2005 and 2015. At each sampling site quantitative samples of macroalgae and associated invertebrate communities were collected by a diver using a standard bottom frame (0.04 m^2^). Samples were sieved in the field on 0.25 mm mesh screens. The residues were stored at −20 °C and subsequent sorting and counting of species was performed in the laboratory using a stereomicroscope. The dry weight of algae was obtained after drying the individuals at 60 °C for 2 weeks. These quantitative data were complemented by information on species occurrence (the HELCOM Red List dataset) (Supplementary Fig. 1).

### Modelled environmental variables

When assessing the effects of climate change on species distribution, ecological understanding is a prerequisite to select the environmental covariates to include in SDM. Therefore, care was taken to include the most relevant ecological variables in order to reach the best projections about the role of climate-driven effects on biotic patterns. When the selection is inadequate, a model may pick up irrelevant variables and its predictive power is low^86^. Earlier studies have shown that water salinity and temperature conditions are anticipated to shape the large scale patterns of benthic macrophyte and invertebrate species in the Baltic Sea area^87^ and these same variables are also expected to change the most in the light of future climate change^38,88,89^.

Modelled temperature and salinity data were produced by the Swedish Meteorological and Hydrological Institute (SMHI) using the echam5/RCAO model run covering the whole Baltic Sea at a grid resolution of 2 NM^89^. In addition to current environmental data, the SMHI datasets had two scenarios for seasonal means: a reference for the years 1978–2007 and the climate scenario A1B for the years 2069–2098. The A1B climate scenario is a scenario proposed in the Special Report on Emissions Scenarios by the Intergovernmental Panel on Climate Change^90^. The A1 scenario group describes a future world of very rapid economic growth, global population that peaks in mid-century and declines after that, and the rapid introduction of new and more efficient technologies. A1B foresees a balance between energy sources, where the balanced energy consumption is defined as not relying too heavily on one particular energy source under the assumption that similar improvement rates apply to all energy supplies and end-use technologies. In our models we used averages of three summer months (June to August) to match our experimental design (see below). The current modelled salinity values were used to divide the Baltic Sea into three regions: entrance (dynamic area between the North and Baltic Seas; salinity > 12), central (the Baltic Proper; salinity 5–12) and marginal regions of the Baltic Sea (inner gulfs; salinity < 5) (Supplementary Fig. 1). These three functionally different regions characterised by genetically distinct and different proportions of marine, brackish and freshwater species^66,91^ were used to define the sites of collection of the experimental organisms and to formalize separate model domains for the spatial predictive modelling (see below) in order to account for the different responses of locally adapted or otherwise differentiated populations.

In addition to the SMHI data layers, depth data acquired from the Baltic Sea Bathymetry Database^92^ were also used as a modeling input variable for *Fucus*.

### Experimental procedures

We quantified the joint effect of salinity and temperature on growth of *Fucus* and survival of *Idotea* in experiments carried out at the Archipelago Sea Research Institute in Seili, SW Finland (60°14′N, 21°58′E) in 2014–2015 (for detailed descriptions of these experiments, see^49,50^). Experimental organisms were collected from multiple populations originating from three functionally different regions defined above (entrance, central and marginal) (Supplementary Fig. 1). *Fucus* was collected by snorkelling on hard bottom habitats at 0.5–2 m from June to July in 2014. To avoid sampling of close relatives we kept a minimum of 5 m distance between sampled algae. *Idotea* was sampled from *Fucus* algae at 1–3 m depth from May to June in 2015. In order to account for among-population variation in tolerance to environmental change, sampling sites were randomly chosen within each region to represent the distributional range of these species along the Baltic Sea salinity gradient.

The collected *Fucus* and *Idotea* were exposed to the current (1978–2007) and future (2069–2098) conditions of surface seawater salinity and temperature (average for the main reproductive period: June to August for *Fucus* and June for *Idotea*; Supplementary Table 2), separately for the three studied regions of the Baltic Sea (Supplementary Fig. 1)^89^. We used aquarium racks with a recirculating water system to provide the appropriate climatic conditions for each of the six treatment combinations of temperature and salinity (i.e. current and future scenario at entrance, central and marginal regions). We started the experiments by putting the organisms in the current conditions of their original region and then adjusted salinity and temperature to match those of the future projections (over the course of 24 h in *Fucus* and 5 days in *Idotea*).

Before deployment, *Fucus* individuals were harvested for apical branches and then half of the apical fronds were exposed to current conditions and the other half to future conditions so that each individual could face both climatic conditions. The apical branches were randomly allocated to replicate 24 l aquaria, each aquarium having 24–35 algae. Within each condition and region we had 12 replicate aquaria, totaling to 72 aquaria and to 2079 algae. For *Idotea*, we only used females as their response is more important for the population growth. *Idotea* were deployed individually in cylindrical cages (length 11 cm, diametre 3.5 cm) inside the aquaria, to allow their monitoring individually. Within each condition and region we had three replicate 54 l aquaria, each aquarium having 30–45 individuals totaling to 468 *Idotea*. *Idotea* were fed with *Fucus* fresh tissue. We monitored the survival and ran the experiments until about a third of the intial number of individuals was left, 140 days for *Fucus* and 55 days for *Idotea*.

During the experiment the survival of *Fucus* was checked on a weekly basis and an individual frond was considered as dead when the thallus showed over 90% of tissue necrosis. The biomass growth of *Fucus* was measured as a difference in algal wet weight between the starting point and the last day of the experiment. The survival of *Idotea* was monitored every two days.

### Modelling environmental responses of *Fucus* and *Idotea* and their tolerance to current climate change

We used hierarchical Bayesian models^93^ and Gaussian processes^35,37^ to study the spatial patterns of *Fucus* and *Idotea* under current and future climate conditions. A rationale for using these methods is that they provide tools to model biologically reasonable joint effects of salinity and temperature. Moreover, they allow us to combine *a priori* biological knowledge with all the information in heterogenous distribution and experimental data as well as to produce posterior distributions from which uncertainty summaries can easily be extracted^94^. We can also explicitly model the spatial autocorrelation in the data not explainable by the covariates^15^.

#### Fucus model

We denote the *Fucus* observations by ${y}_{i}$, where ${y}_{i}=\varnothing$ corresponds to the absence of *Fucus* in the survey data and the death of *Fucus* within the study period in the experimental data, and where ${y}_{i}\in {\mathbb{R}}$ corresponds to a logarithmic estimate of the biomass (survey data) or growth of *Fucus* (experimental data), given it is present, in the $i\,$th observation. In order to cover both presence/absence and biomass or growth observations, we built a hurdle model so that

1 $$p({y}_{i}|{\pi }_{i},{\mu }_{i},{\tau }_{i}^{2})=\{\begin{array}{c}(1-{\pi }_{i})\\ {\pi }_{i}\times N({y}_{i}|{\mu }_{i},{\tau }_{i}^{2})\end{array}\begin{array}{c}\,\mathrm{if}\,{y}_{i}=\varnothing \\ \,\mathrm{if}\,{y}_{i}\in {\mathbb{R}},\end{array}$$

where ${\pi }_{i}$ is the probability of occurrence of *Fucus* and $N({y}_{i}|{\mu }_{i},{\tau }_{i}^{2})$ is the Gaussian probability density function with mean ${\mu }_{i}$ and variance ${\tau }_{i}^{2}$. We used one variance parameter, ${\tau }_{{\rm{exper}}}^{2}$, for all experimental observations and another variance parameter, ${\tau }_{{\rm{dist}}}^{2}$, for all distribution observations. For ease of implementation we standardized both data to have zero mean and standard deviation of one before estimating the model parameters. Since we follow the Bayesian approach we gave the variance parameters a prior distribution. We used a half Student-*t* distribution with mean zero and scale 1 which gives weak prior preference for small variances but allows also the data to be explained as pure noise.

In the case of distribution data, we modelled the mean of the Gaussian distribution, ${\mu }_{i},$ and the logarithm of the odds ratio of the occurrence probability, ${{\rm{logit}}}^{-1}({\pi }_{i})=\,\mathrm{log}(\frac{{\pi }_{i}}{1-{\pi }_{i}})$, as functions of salinity, temperature, depth and spatial location so that, e.g., ${\mu }_{i}=\alpha +{f}_{{\rm{ST}}}({x}_{{\rm{S}},i},{x}_{{\rm{T}},i})+{f}_{{\rm{D}}}({x}_{{\rm{D}},i})+{\rm{\varphi }}({s}_{i})$. We will denote these additive models for ${{\rm{logit}}}^{-1}({\pi }_{i})$ and ${\mu }_{i}$ respectively as *occurrence and biomass models*. Here,$\,\alpha$ is the intercept, which was given a vague $N(0,10)$ prior, ϕ is the spatial random effect, ${f}_{{\rm{ST}}}$ is the response function along temperature, ${x}_{{\rm{T}}}$, and salinity, ${x}_{{\rm{S}}}$, and ${f}_{{\rm{D}}}$ is the response function along depth, ${x}_{{\rm{D}}}$. The depth function accounts for different light, nutrient and wave force conditions in sampling sites and the spatial random effect accounts for spatially correlated overdispersion in the data, which causes nearby areas to have similar biomasses because of, for example, associations unexplained by the available covariates^5,6,15^. The depth function and spatial random effect were set to zero for all experimental observations.

*Fucus* and *Idotea* have a lower physical tolerance limit to salinity at around 4 and 3, respectively^41,95^; above these thresholds survival and growth rise and level-off at a saturation point^49,50,53^. Since *Fucus* and *Idotea* are boreal species and they do not like either too cold or warm environments^96^ the response along temperature is positive at low temperatures and negative at large temperatures with optimal summer temperature at around 14–20 degrees^53,95^. However, the exact functional form of the responses is unknown and it is likely that temperature and salinity have also joint effects^47,48^. Hence, constructing a parametric model for the effects of salinity and temperature would be challenging. For this reason we modeled them using semi-parametric GP models. A GP is a stochastic process that defines probability distribution for functions. It is defined by a mean function and a covariance function, which determine the *a priori* assumptions on properties of the process^97^; such as how large and how fast (smoothness) the responses along temperature and salinity are. The exact form of the response function is then estimated from the data by solving the posterior of the functions using the Bayes theorem^9,35,37^.

We gave the GP prior of ${f}_{{\rm{ST}}}$ zero mean and Gaussian covariance function

$$\begin{array}{c}{\rm{Cov}}({f}_{{\rm{ST}}}({x}_{{\rm{S}},i},{x}_{{\rm{T}},i}),\,{f}_{{\rm{ST}}}({x}_{{\rm{S}},j},\,{x}_{{\rm{T}},j}))={\sigma }_{{\rm{ST}}}^{2}{{\rm{e}}}^{-{r}_{ij}^{2}}\,{\rm{where}}\\ {r}_{ij}=\sqrt{{({x}_{{\rm{T}},i}-{x}_{{\rm{T}},j})}^{2}/{\lambda }_{{\rm{T}}}^{2}-{({x}_{{\rm{S}},i}-{x}_{{\rm{S}},j})}^{2}/{\lambda }_{{\rm{S}}}^{2}}\end{array}$$

is the scaled Euclidean distance between salinity-temperature locations, ${\sigma }_{{\rm{ST}}}^{2}$ is the variance parameter governing the magnitude of the effect and $\lambda$ are the length-scale parameters that govern the smoothness of responses along temperature and salinity. The variance and length-scale parameters were estimated so we gave priors for them. We used a half Studen*t*-*t* distribution with mean zero and scale one for ${\sigma }_{{\rm{ST}}}$ which gives equal prior weight to temperature-salinity effect as to the random variation in (1). In order to avoid too flexible response functions the length-scales should be of the same order of magnitude as the range of covariates^37^. The modeled salinity range is from 0 to 30 and the modeled temperature range is from 12 to 20 degrees. Hence, we gave the length-scales log-Gaussian prior distributions with location and scale parameters such that 90% of the prior probabilities were included within the salinity range 11–28 with median of 18 and the temperature range 5.5–13.5 degrees with median of 8.5 degrees. This ensures that the *a priori* plausible functions can change rapidly but do not have many modes within the study limits (See Supplement). Moreover, in order to include the *a priori* qualitative information that ${f}_{{\rm{ST}}}$ should be increasing with increasing salinities greater than 5, and that it should be decreasing with increasing temperatures above 20 degrees, we set constraints to the derivatives of ${f}_{{\rm{ST}}}$. These biologically realistic constraints were encoded into the model by virtual derivative observations^98^. We constructed two lattice grids, one that covered the modeled temperature range and salinities from 5 to 25 and another that covered the modeled salinity range at 24 degrees temperature. At the nodes of the former lattice we imposed positive derivative constraint along salinity by defining a probit likelihood function for the derivative of the latent function ${\rm{\Phi }}(\partial {f}_{{\rm{ST}}}/\partial {x}_{S})$, where ${\rm{\Phi }}(\,\cdot \,)$ is the standard Gaussian cumulative distribution function^98^. At the nodes of the latter lattice we imposed negative derivative constraint along temperature with likelihood ${\rm{\Phi }}(\,-\,\partial {f}_{{\rm{ST}}}/\partial {x}_{{\rm{T}}})$. The resolution of the lattices was set so that they impose the monotonicity constraint with length-scales above their *a priori* plausible values. See Supplementary Fig. 2 for illustration.

We modeled also the effect of depth on *Fucus* occurrence probability and biomass using a GP with zero mean and Gaussian covariance function. The spatial random effect was modeled with a spatial GP with zero mean and an exponential covariance function,

$${\rm{Cov}}({{\rm{\varphi }}}_{{\rm{\varphi }}}({s}_{i}),{{\rm{\varphi }}}_{{\rm{\varphi }}}({s}_{j}))={\sigma }_{{\rm{\varphi }}}^{2}{{\rm{e}}}^{-\sqrt{{\Sigma }_{d=1}^{2}{({s}_{d,i}-{s}_{d,j})}^{2}/{\lambda }_{{\rm{\varphi }}}^{2}}}.$$

We used again a half Student-*t* distribution with mean zero and scale one for the standard deviation parameters. In order to give more weight to smooth unimodal functions, the length-scale parameter of the depth effect was given a Gaussian prior with mean five meters and standard deviation 1. The inverse of the spatial length-scale was given a half Student-*t* prior which gives more weight to smooth spatial random effects and in the absence of spatial autocorrelation shrinks the spatial random effect to constant.

#### Idotea model

With *Idotea* we analyzed only its occurrence probability. The occurrence models were otherwise the same as the occurrence models for *Fucus* but the depth response function was replaced by the effect of *Fucus* biomass, ${x}_{{\rm{F}}}$. *Fucus* is amongst the most widespread habitat and food for *Idotea* on hard bottoms (e.g.^54^). Hence, in the absence of *Fucus* the probability of occurrence of *Idotea* can be lower than in the presence of *Fucus* and with elevating *Fucus* biomass the probability is expected to increase to a threshold above where further changes are marginal. Hence, we modeled the effect of *Fucus* biomass as a sum of delta and Michaelis-Menten (type II,^99^ functions ${f}_{{\rm{F}}}({x}_{{\rm{F}}})={\alpha }_{{\rm{F}}}\delta ({x}_{{\rm{F}}} > 0)+{\beta }_{{\rm{F}}}{x}_{{\rm{F}}}/({c}_{{\rm{F}}}+{x}_{{\rm{F}}})$, where $\delta ({x}_{{\rm{F}}} > 0)$ is a delta function that is one if ${x}_{{\rm{F}}} > 0$ and zero otherwise, and ${\alpha }_{{\rm{F}}}$ is the effect of presence of *Fucus*; the weight ${\beta }_{{\rm{F}}}$ defines the asymptotic effect and the half saturation parameter, ${c}_{{\rm{F}}}$, is the *Fucus* biomass where the effect of the Michaelis-Menten response has reached the half of its maximum. We used vague $N(0,10)$ priors for ${\alpha }_{{\rm{F}}}$, ${\beta }_{{\rm{F}}}$ and ${c}_{{\rm{F}}}$.

#### Combining experimental and distribution data

The physical tolerance of *Fucus* and *Idotea* to temperature and salinity is expected to be the same in experimental and natural conditions. Hence, when analyzing distribution and experimental data together we assumed that the functional form of the response along temperature and salinity, ${f}_{{\rm{ST}}}$, should be the same in both cases. However, the magnitude of the response might vary between experiments and observational data. These assumptions were encoded into the model by using ${f}_{{\rm{ST}}}$ directly as the response function in the experiments and scaling it by positive constant $c$ in the distribution data, that is ${f}_{{\rm{ST}},{\rm{dist}}}=c{f}_{{\rm{ST}}}$ (Fig. 1). In practice, this induces a zero mean multivariate Gaussian process prior for a vector valued function ${\tilde{f}}_{{\rm{ST}}}=[{f}_{{\rm{ST}}},{f}_{{\rm{ST}},{\rm{dist}}}]$ with cross covariance function.

$${\rm{Cov}}({f}_{{\rm{ST}}}({x}_{{\rm{S}},i},{x}_{{\rm{T}},i}),{f}_{{\rm{ST}},{\rm{dist}}}({x}_{{\rm{S}},j},{x}_{{\rm{T}},j}))={\sigma }_{{\rm{ST}},{\rm{exper}}}{\sigma }_{{\rm{ST}},{\rm{dist}}}{{\rm{e}}}^{-{({x}_{{\rm{T}},i}-{x}_{{\rm{T}},j})}^{2}/{\lambda }_{{\rm{T}}}^{2}-{({x}_{{\rm{S}},i}-{x}_{{\rm{S}},j})}^{2}/{\lambda }_{{\rm{S}}}^{2}}$$

where ${\sigma }_{{\rm{ST}},{\rm{exper}}}$ and ${\sigma }_{{\rm{ST}},{\rm{dist}}}$ are the marginal standard deviations of the experimental and distribution data responses. Hence, the correlation between these two GPs is one but they have different marginal variances. The rest of the model components were mutually independent between the experimental and distribution data. When predicting climate change scenarios, the shared response function allows information flow from the experiments to the projections concerning natural conditions. The extra variation in the observational data is then modeled by the spatial random effect and the response along depth (*Fucus* biomass in the *Idotea* model) and the mutually independent intercept parameters allow for different overall prevalence in these data sets.

#### Model estimation and validation

Since the absence observations in *Fucus* model (1) are not covered by the Gaussian distribution, the *Fucus* model factorizes with respect to the occurrence/survival model and biomass/growth model. Hence, we estimated independently *Fucus* occurrence/survival and biomass/growth models and *Idotea* occurrence model for experimental and distribution data separately and for combined data. The model components (the intercept, effects of salinity-temperature, depth and *Fucus*, as well as the spatial random effect) can be represented as GPs or Gaussian random variables. Hence, conditional on parameters of covariance functions and the half saturation parameter (*hyperparameters* from this on), our model is a GP SDM with additive covariance function, see^15^, and their supplement for details). We estimated the hyperparameters with their *maximum a posterior estimate* (MAP; the value maximizing their marginal posterior density) and calculated the posterior distributions of the response functions conditional on the MAP estimate of hyperparameters. The MAP estimate was searched with gradient based optimization of the marginal posterior of hyperparameters. We used the expectation propagation algorithm to construct approximations for the marginal posterior of hyperparameters. All the models were implemented by using the GPstuff toolbox^100^. For technical details on the inference method see the work of^15^ and the documentation of the GPstuff toolbox.

The models were used to study the responses along the covariates by drawing the relative change in biomass and occurrence probability along covariates compared to average biomass and 0.5 probability respectively^15^ (Fig. 2 and Supplementary Fig. 3). We used the models also to predict (i.e., calculate the posterior predictive probability of) the occurrence of *Idotea* and the biomass of *Fucus* across the Baltic Sea under the current and the future climate scenario at lattice grid with a cell size of 2 NM. Moreover, the projections were made separately for representative salinity and temperature values of three functionally different sub-regions of the Baltic Sea (Figs 1 and 3 and Supplementary Figs 1 and 4**)**.

We analyzed models’ explanatory power by calculating the coefficient of determination (R^2^) for the posterior mean of biomass/growth models and the Tjur R^2^ summary statistics^101^ for the occurrence models. Tjur R^2^ is a coefficient of discrimination defined as the difference in the mean occurrence probability predicted for occupied versus unoccupied sampling units. We calculated the proportion of variation in models’ posterior mean projections for training data explained by different components by partitioning the total variation to its components as described in a supplement of reference^13^. We also validated and compared the models using independent test data. To test model performance in predicting *Idotea* and *Fucus* distribution under current conditions we used all experimental data and 2000 randomly sampled distribution data points for model estimation and used the rest of the distribution data samples (n = 4407) to compare the goodness of projections. Hence, the training and test sets spanned the same covariate range which corresponds to typical species distribution predictions under current conditions. To test model performance in predicting future conditions we split the distribution data at 17.5 degrees temperature and used the experimental data and distribution data below 17.5 degrees in the training set (n = 904) and distribution data above it in the test set (n = 2527). In this case training and test data span totally different covariate ranges which mimics species distribution projections under very drastic environmental change. In both tests, we calculated the percentage of true presence/absence classifications for each of the occurrence models and the root mean squared error (RMSE) in the case of the biomass models.

## Supplementary information

Supplementary information

## Data Availability

The datasets that were generated and/or analysed during the current study are freely available from the corresponding author on a request.
