# Supplementary material for: Integrating experimental and distribution data to predict future species patterns
Source: Sci Rep. 2019 Feb 12;9:1821. doi: 10.1038/s41598-018-38416-3 (PMC6372580; doi:10.1038/s41598-018-38416-3)
Supplement: Supplementary file 1 — Supplementary information [file 41598_2018_38416_MOESM1_ESM.pdf]

ONLINE MATERIAL FOR

Integrating experimental and distribution data to predict future species patterns

Jonne Kotta[1*], Jarno Vanhatalo[2], Holger Jänes[1,3], Helen Orav-Kotta[1], Luca Rugiu[4], Veijo Jormalainen[4], Ivo Bobsien[5], Markku Viitasalo[6], Elina Virtanen[6], Antonia Nyström Sandman[7], Martin Isaeus[7], Sonja Leidenberger[8], Per R Jonsson[9] & Kerstin Johannesson[9]

[1]Estonian Marine Institute, University of Tartu, Mäealuse 14, EE-12618 Tallinn, Estonia
[2]Department of Mathematics and Statistics and Faculty of Bio- and Environmental Sciences, University of Helsinki, FIN-00014 Helsinki, Finland
[3]Centre for Integrative Ecology, Deakin University, 221 Burwood Hwy, Melbourne, Victoria 3125 Australia
[4]Department of Biology, University of Turku, FIN-20014 Turku, Finland
[5]GEOMAR Helmholtz Centre for Ocean Research Kiel, 24105 Kiel, Germany
[6]Finnish Environment Institute, FIN-00251 Helsinki, Finland
[7]AquaBiota Water Research, Löjtnantsgatan 25, SE-11550 Stockholm, Sweden
[8]Ecological Modelling Group, School of Bioscience, University of Skövde, SE-54128 Skövde, Sweden
[9]Department of Marine Sciences – Tjärnö, University of Gothenburg, Tjärnö, SE-45296 Strömstad, Sweden

*corresponding author, email: jonne@sea.ee

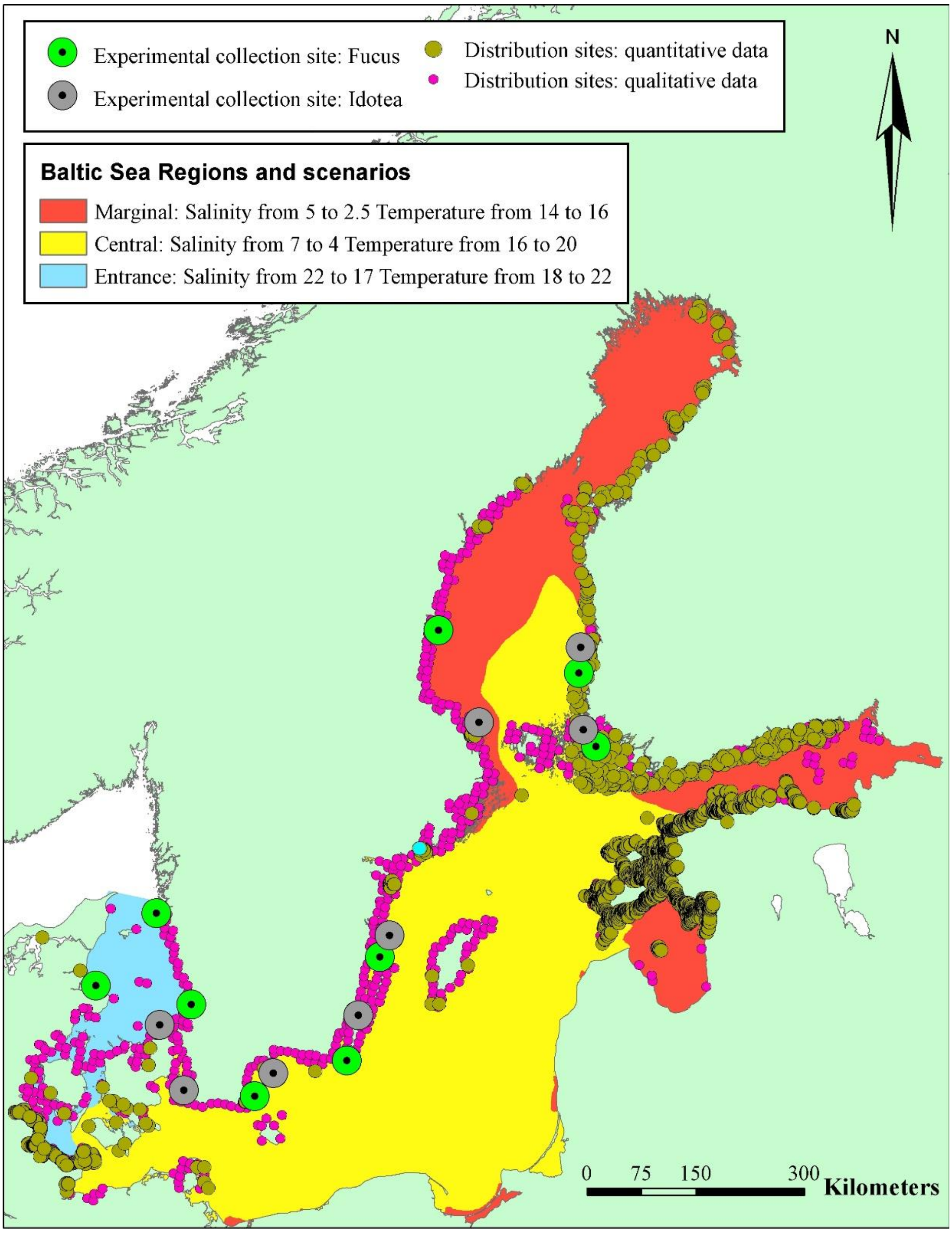

Supplementary Figure 1. Map of study area. Smaller dots indicate survey sites and larger dots indicate sites where experimental plants and invertebrates were collected. Quantitative data refers to sampling stations where biomasses of species were quantified and qualitative data refers to sampling stations where only species presences/absences were recorded. Areal colours depict different regions of the Baltic Sea assessed by surface seawater salinities of the SMHI echam5/RCAO model under current climatic conditions: entrance (dynamic area between the North and Baltic Seas; salinity >12), central (the Baltic Proper; salinity 5−12) and marginal regions of the Baltic Sea (inner gulfs; salinity <5). Each region is assigned its current (1978−2007) and future (2069−2098) scenario of surface seawater salinity and temperature (average of June to August) that was used to define treatment levels in the *Fucus* and *Idotea* tolerance experiments.

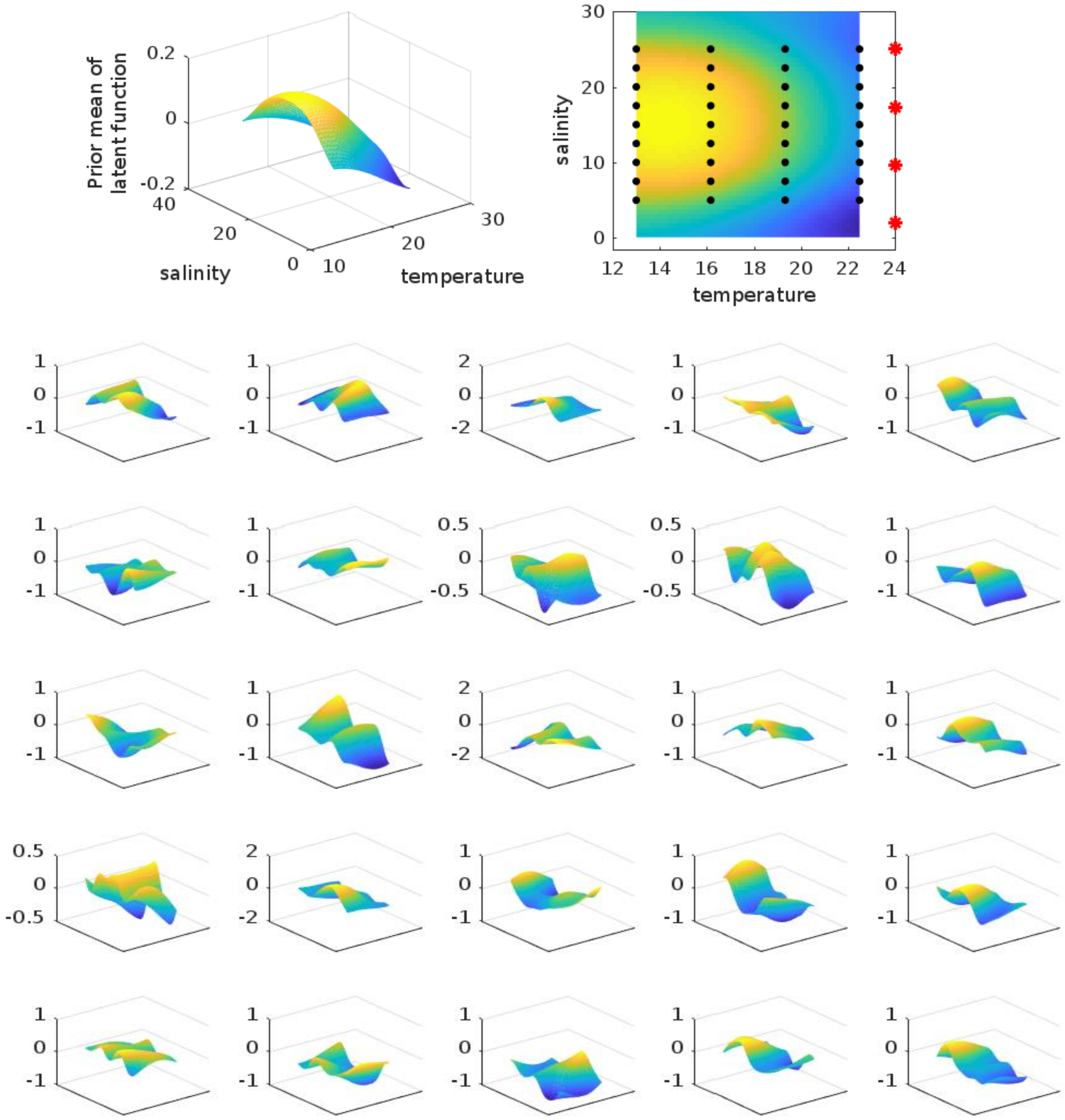

Supplementary Figure 2. *The plots at the top row* show the prior mean (on the left) of the latent function $f_{\mathrm{ST}}(x_{\mathrm{S},i}, x_{\mathrm{T},i})$ and the locations of virtual observations that were used to impose the prior monotonicity constraints (on the right). Black dots show the locations where we imposed positive derivative constraint along salinity, $\Phi(\partial f_{\mathrm{ST}}/\partial x_S)$, and the red dots show the locations where we imposed negative derivative constraint for prior mean along temperature $\Phi(-\partial f_{\mathrm{ST}}/\partial x_{\mathrm{T}})$. *The five lower rows* show random draws from a Gaussian process (GP) prior for $f_{\mathrm{ST}}(x_{\mathrm{S},i}, x_{\mathrm{T},i})$ with this prior mean constraint. As seen from the plots, the possible realizations from the GP are flexible and the prior monotonicity constraint is very moderate.

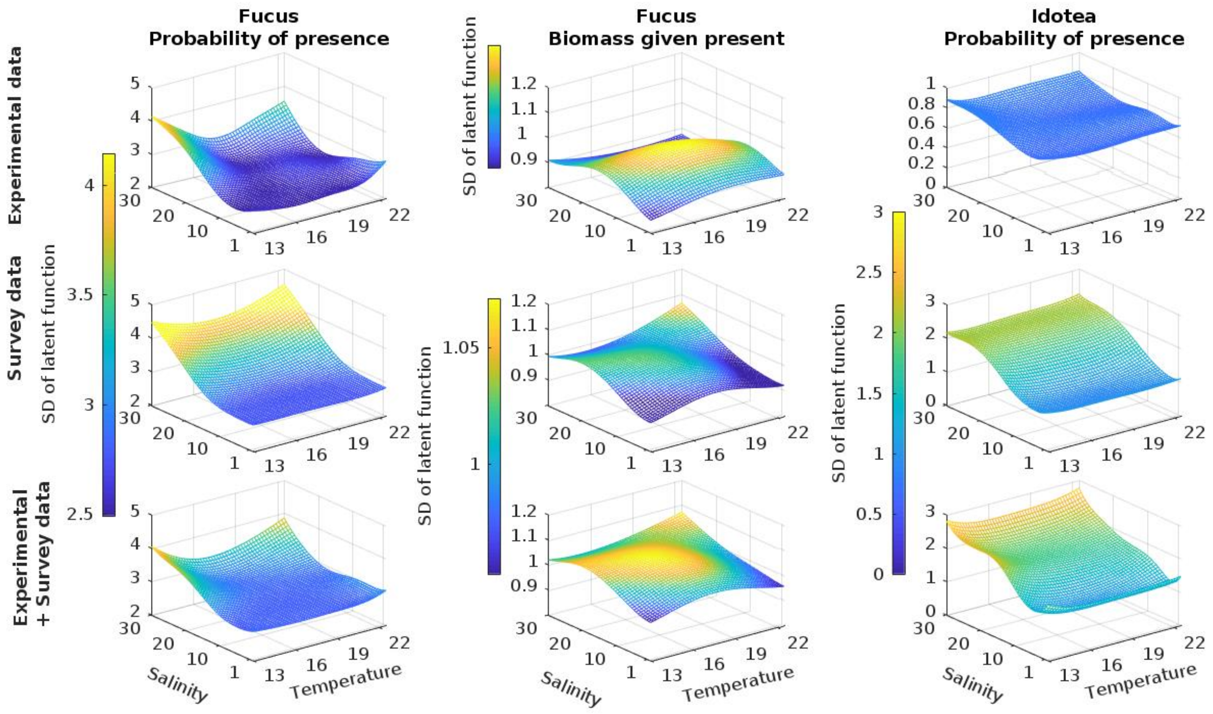

Supplementary Figure 3. Posterior uncertainty in the response functions along salinity and temperature. Each surface plot shows the posterior standard deviation of the latent function $f_{\mathrm{ST}}(x_{\mathrm{S},i}, x_{\mathrm{T},i})$. The latent function corresponds to log odds ratio of change in probability of presence and log relative change in growth (experiment) or biomass (survey and combined model). In general, the variance is smallest at regions of salinity-temperature values from where we have most observations (low salinity and low temperature) and increases in regions with less data (high salinity values).

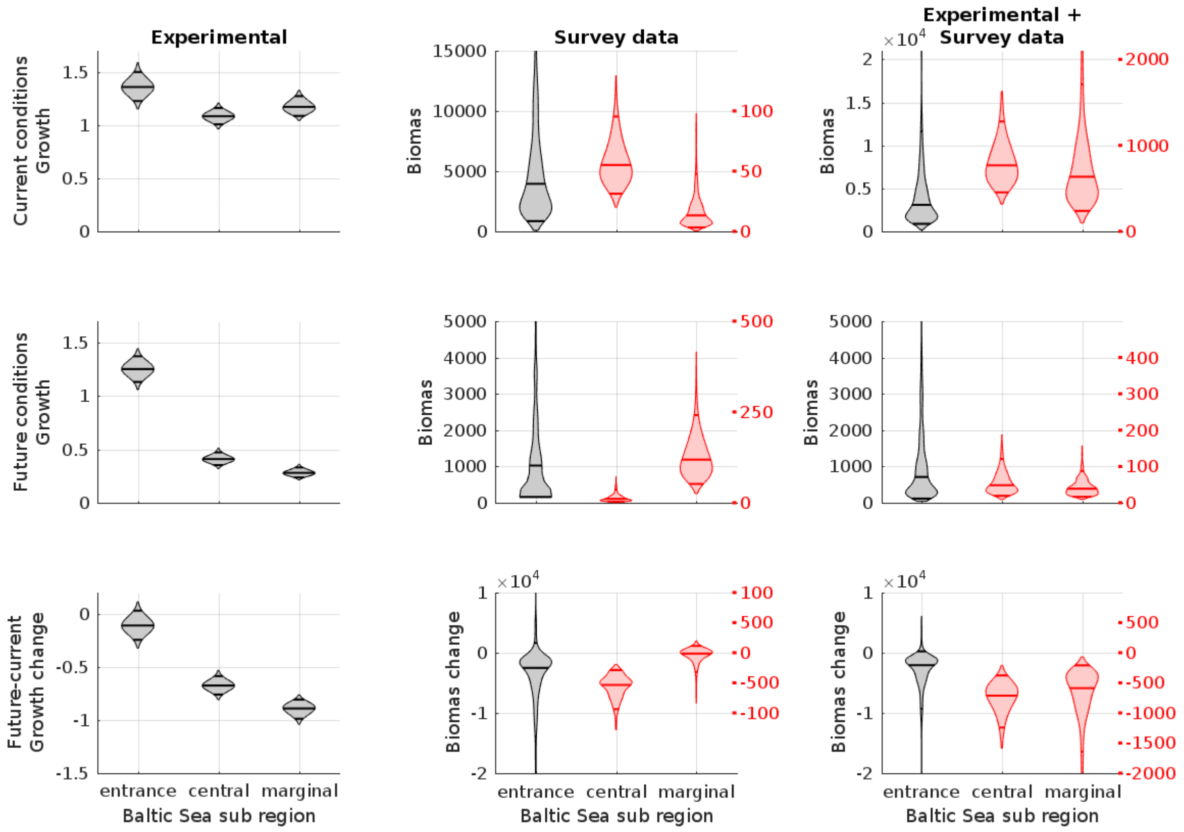

Supplementary Figure 4. The effect of climate change to the mean growth (experimental data, relative increment of initial biomass value) or biomass (survey data, g m^{-2}) of *Fucus* in three regions of the Baltic Sea. Here mean biomass equals probability of presence multiplied by biomass conditional on presence; mean growth equals probability of survival multiplied by growth rate; that is $\pi \times \mu$. The first and second row show the mean biomass or growth rate under current and future climate. The third row shows their difference (future – current). In each mark lines show the posterior expectation and the 95% confidence interval and the shape of the mark indicates the shape of the posterior distribution.

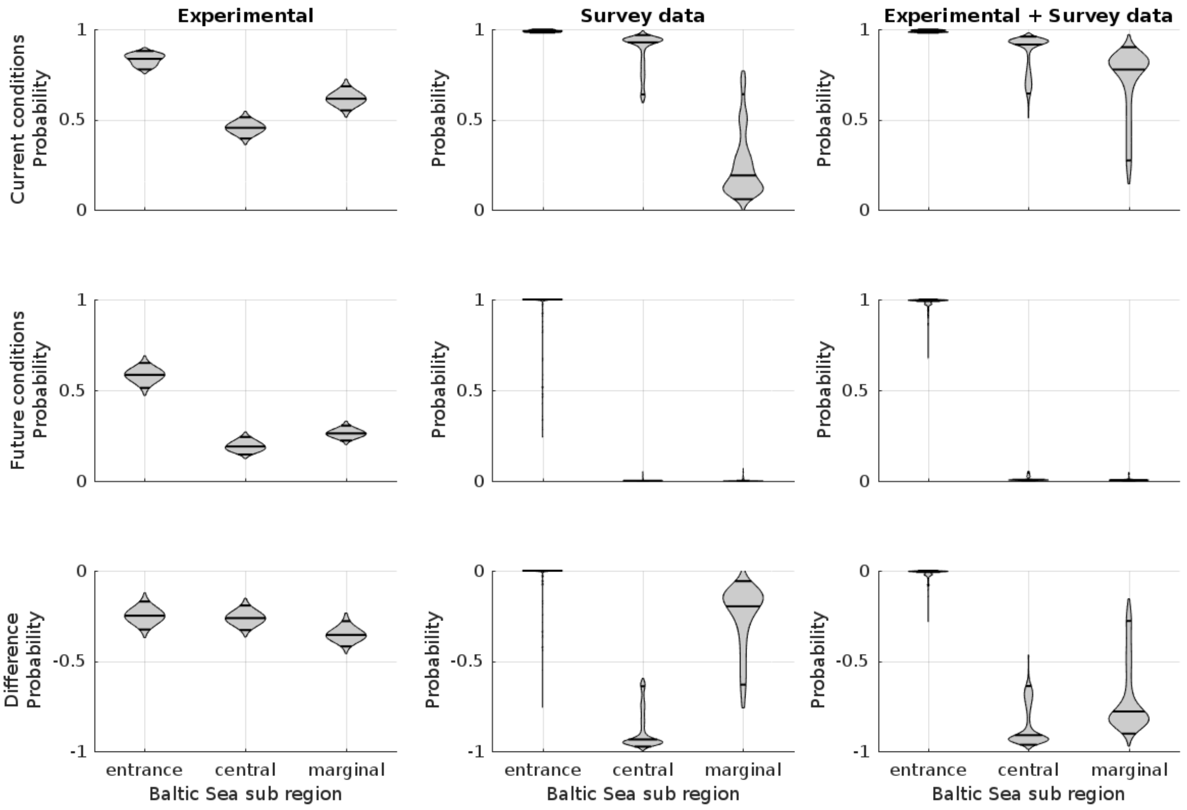

Supplementary Figure 5. The effect of climate change to the mean probability of presence (survey data) or the mean probability of survival of *Idotea* (experimental data) in three regions of the Baltic Sea. The first and second row show the mean probabilities under current and future climate. The third row shows their difference (future – current). In each mark, lines show the posterior expectation and the 95% confidence interval and the shape of the mark indicates the shape of the posterior distribution.

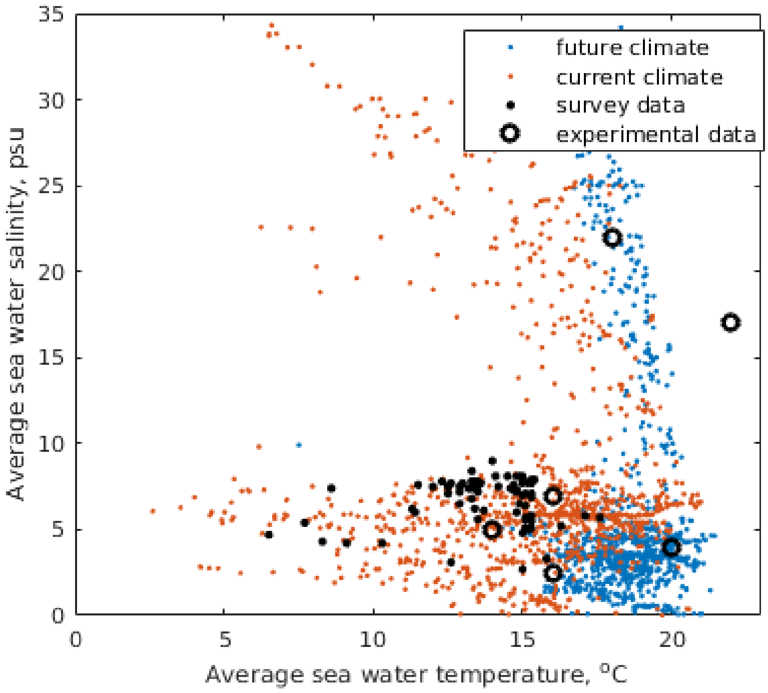

Supplementary Figure 6. Illustration of the extrapolation task in our study. Blue and red dots correspond to the projected future and current temperature and salinity values at 1000 randomly chosen locations in our study region the Baltic Sea (predictions based on climate scenarios). Black dots and circles show temperature and salinity values in a subset of the distribution data and in the experimental data respectively. The Baltic Sea environment is expected to be warmer and less saline and therefore the environmental conditions are expected to move towards the lower right corner of the covariate space from where we do not have distribution data.

Supplementary Table 1. Models' predictive performance. Predictive performance is measured by percentage of correct presence predictions (PCC, occurrence models) and root mean squared error (RMSE, biomass models). Interpolation tests are done by training models with experimental data and randomly sampled distribution data (n=2000) and predicting for randomly sampled test distribution data (n=4407). These evaluate goodness of current conditions predictions. Extrapolation test is done by training models with experimental data and distribution data below 17.5 degrees temperature (n=904) and predicting for distribution data above 17.5 degrees temperature (n=2527). These evaluate goodness of predictions for conditions not yet experienced in the nature.

		**Interpolation**		**Extrapolation**	
**Species**	**Data source (model)**	**PCC**	**RMSE**	**PCC**	**RMSE**
Fucus	Experiments	0.60		0.74	
	Distribution	0.80	0.88	0.74	0.94
	Distribution + Experiments	0.80	0.88	0.76	0.92
Idotea	Experiments	0.72		0.82	
	Distribution	0.84		0.84	
	Distribution + Experiments	0.84		0.84	

Supplementary Table 2. Average summer sea surface temperature (SST) and salinity (SSS) used for current and future conditions within the three regions, separately for the experiments in *Fucus* and *Idotea*. For *Fucus*, current conditions were calculated by averaging the monthly means from June to August, and for *Idotea*, by averaging the mean conditions for June. Data for current conditions was obtained from the Baltic Nest Institute (http://www.balticnest.org) and those for the future expected conditions were set according to the model of Meier et al. (2012). The differences in conditions for *Fucus* and *Idotea* are due to both the difference in the length of the main reproductive period used in averaging and the difference in the locations of the sampled populations within the entrance region.

**Species**	**Region**	**Current**		**Future**	
		SSS (PSU)	SST (°C)	SSS (PSU)	SST (°C)
Fucus	Entrance	22	18	17	21
	Central	7	16	4	20
	Marginal	5	14	2.5	16
Idotea	Entrance	12	14	7	17
	Central	7	14	4	18
	Marginal	5	11	2.5	14
